# Moxibustion for uterine contraction pain: A protocol for systematic review and meta-analysis

**DOI:** 10.1097/MD.0000000000032195

**Published:** 2022-12-02

**Authors:** Xuewei Zhao, Baiyan Liu, Qi Zhang, He Wang, Yu Tian, Fuchun Wang

**Affiliations:** a Department of Acupuncture and Tuina, Changchun University of Chinese Medicine, Changchun, China; b Rehabilitation College, Hangzhou Medicine College, Hangzhou, China.

**Keywords:** meta-analysis, moxibustion, protocol, systematic review, uterine contraction pain

## Abstract

Uterine contraction pain is a common postpartum condition that often plagues mothers and can interfere with feeding and normal life for young children. Many investigations have demonstrated that moxibustion has certain advantages in the treatment of uterine contraction pain, but the effectiveness, safety, and advantages of various methods have not been confirmed by high-quality meta-analyses. This study will conduct a systematic review and meta-analysis to evaluate the safety and effectiveness in moxibustion, in order to provide a reference for further clinical treatment for uterine contraction pain.

**Methods:**

Nine electronic databases, including PubMed, Medline, Embase, Web of Science, Cochrane Central Register of Controlled Trials, China National Knowledge Infrastructure, Chinese Biomedical Literature Database, Chinese Scientific Journal Database, Wan-Fang Database and 1 clinical trial register platforms: ClinicalTrials.gov (www.ClinicalTrials.gov/) will be searched using English and Chinese search strategies. All eligible studies are randomized controlled trials of moxibustion treatment for uterine contraction pain, published on or before December 31, 2021. The screening process will be developed by 2 independent authors, and network meta-analysis will be performed with RevMan (V5.3) software.

**Results:**

This study will provide a high-quality review that will be used to evaluate the safety and effectiveness of moxibustion for the treatment of uterine contraction pain.

**Conclusion:**

The results of this study will provide evidence to support whether moxibustion can effectively intervene in uterine contraction pain.

## 1. Introduction

Severe pain in the lower abdomen caused by strong contractions of the uterus during delivery is known as uterine contraction pain^[1]^ and occurs 1 to 2 days after delivery of the fetus and placenta, and the pain usually disappears after 2 to 3 consecutive days.^[2]^ Uterine contraction pain is one of the common postpartum complications,^[3]^ and most women can be tolerated, but due to breastfeeding the baby is caused by reflex contractions increase,^[4]^ accompanied by sweating, nausea and vomiting, reduced milk, etc. resulting in poor rest and sleep, and the postpartum life of the mother is affected by the prolonged period of time.^[5]^ Some studies have reported that the incidence of uterine contraction pain is 30% to 40% among women in labor,^[6]^ and 4% to 10% of women in normal labor may experience chronic pain.^[7]^ Uterine contractions are associated with the elimination of malignant fluid and regeneration of the uterus. As the global population is gradually decreasing, women’s fertility and health are highly valued, and uterine contraction is a necessary part of every woman’s life, so uterine contraction pain should be taken seriously.

Nowadays, researchers^[8–10]^ are showing some interest in complementary and alternative therapies for uterine contraction pain because of the risks associated with conventional medications. Several randomized controlled trials have shown the advantages of moxibustion in the treatment of uterine contraction pain, especially in terms of safety and side effects.^[11,12]^ Therefore, this study will conduct a meta-analysis to investigate the effectiveness, safety, and advantages of moxibustion and compare the differences between them to provide a basis for clinical treatment.

## 2. Methods and analysis

Our protocol should be reported basing on the Preferred Reporting Items for Systematic Reviews and Meta-Analyses Protocols statement guidelines.^[13]^ And has been registered in the PROSPERO International Prospective Registry of Systematic Reviews (CRD42022372169).

### 2.1. Inclusion criteria

#### 2.1.1. Types of studies.

Only randomized controlled trials on the moxibustion for uterine contraction pain published on or before December 31, 2021 will be included in this study. All eligible studies languages will be limited to Chinese and English, but no limited to countries and publication status.

#### 2.1.2. Types of patients.

Women were required to be 18 years of age or older, have uterine contraction pain, and have a smooth labor with no other postpartum complications or comorbidities.

#### 2.1.3. Types of intervention.

The treatment group used moxibustion, including multiple moxibustion methods alone or in combination with other methods, and the control group used interventions excluding moxibustion (such as western medicine, placebo moxibustion, etc.) We had no restrictions on the modality or duration of the intervention.

#### 2.1.4. Outcomes.

Outcomes include efficacy and safety. The primary outcome measure will be the degree of clinical symptom relief. Include at least 1 visual analogue scoring scale or World Health Organization pain grading scale. Additional outcomes will be duration of malabsorption, uterine regeneration, and degree of postpartum recovery.

### 2.2. Database search strategy

The search strategy will be based on the Cochrane handbook guidelines (5.1.0). We will search 9 electronic databases, including PubMed, Medline, Embase, Web of Science, Cochrane Central Register of Controlled Trials, China National Knowledge Infrastructure, Chinese Biomedical Literature Database, Chinese Scientific Journal Database and Wan-Fang Database to identify literature of moxibustion for uterine contraction pain, and the search period is from inception to December 31, 2021. Besides, we will also search 1 clinical trial register platforms: ClinicalTrials.gov (www.ClinicalTrials.gov/) for in-progress trials with unpublished data. The search used a combination of subject words and free words, and the search strategy was determined after multiple presearches. The search terms included such as moxibustion, uterine contraction pain, treatment, randomized. The detailed search strategy is presented in Table 1.

**Table 1:** Search strategy for PubMed.

No	Search terms
#1	uterine contraction [MeSH Terms]
#2	unterine contraction pain [Title/Abstract] OR post-partum uterine contraction pain [Title/Abstract] pain of uterine contraction [Title/Abstract]
#3	#1OR #2
#4	moxibustion [MeSH Terms]
#5	heat-sensitive moxibustion [MeSH/Abstract] OR thunder-fire moxibustion [MeSH/Abstract]
#6	#4 OR #5
#7	Randomized Controlled Trial [Publication Type]
#8	Randomized [Title/Abstract] OR Randomized Controlled Trial [Title/Abstract] OR Randomly [Title/Abstract]
#9	#7 OR #8
#10	#3 AND #6 AND #9

### 2.3. Data collection and analysis

#### 2.3.1. Selection of studies.

**Figure 1.**
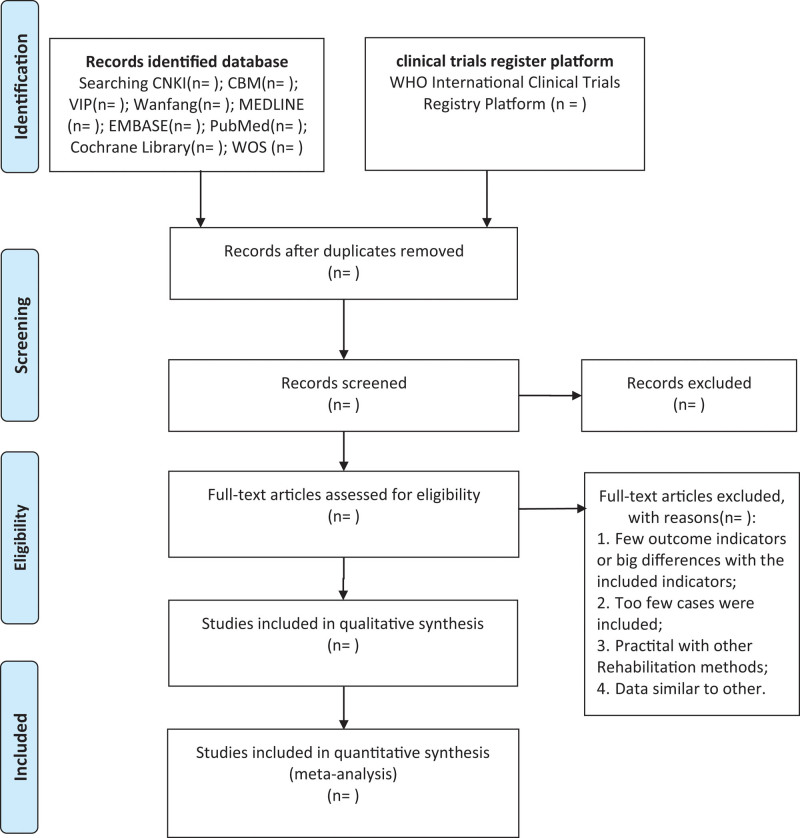
PRISMA flow diagram of study and exclusion. CBM = Chinese Biomedical Literature Database, CNKI = China National Knowledge Infrastructure, PRISMA = Preferred Reporting Items for Systematic Reviews and Meta analyses, VIP = Chinese Scientific Journal Database, WHO = World Health Organization.

After completing all the search work, the results will be exported to Endnote software Version X9, and repetitive studies will be deleted by the software. The process of filtering documents will be completed independently by the 2 reviewers and then the eligible literature will be selected separately based on the screening criteria. When differences arise at any stage, we will invite a third reviewer to discuss arbitration. The studies excluded after reading the full text will also be documented and explained why they were excluded. The research flow chart is shown in Figure 1.

#### 2.3.2. Data extraction and management.

Two reviewers will independently select literature and extract data in accordance with the retrieval strategy, and the results will be cross-matched. They will make an Excel to extract literature data, which includes fundamental information (research title, first author, sample size, age, year, course of disease, treatment period), intervention information (such as treatment method, course of treatment, comparison group, etc.), key elements of bias risk evaluation and outcome indicators. If the differences encountered cannot be resolved through discussion, a third reviewer will be invited to resolve them.

### 2.4. Assessment of risk of bias and reporting of study quality

Two independent reviewers will assess the risk of bias with Cochrane Risk of Bias Tool according to the Cochrane Handbook 5.1.0 for Systematic Reviews of Interventions. The 2 reviewers will assess each included studies from 7 dimensions, which consist of the risk of bias of sequence generation, allocation concealment, blinding of participants personnel and outcome assessment, incomplete outcome data, selective outcome reporting and other bias. At last, the assessment results will be divided into 3 levels: low risk, high risk, and uncertain risk. If differences arise during the assessing process, they will be resolved through intragroup discussions, contacting authors to determine details with the third-party arbitrator.

### 2.5. Measures of treatment effect

The enumeration data were expressed as relative risk, the measurement data adopted mean difference, and each effect amount was expressed in a 95% confidence interval.

### 2.6. Dealing with missing data

We will e-mail the corresponding author to obtain the necessary information, which is missing or insufficient. If failed, the analysis will be conducted based on the available studies, and we will review the potential impact of missing information.

### 2.7. Assessment of heterogeneity

Heterogeneity assessment depends on *I*^2^. *I*^2^ < 25% means negligible heterogeneity, 25% ≤ *I*^2^ < 50% means mild heterogeneity, 50% ≤ *I*^2^ < 75% means moderate heterogeneity, and *I*^2^ ≥ 75% means high heterogeneity.^[14]^

### 2.8. Assessment of reporting biases

Funnel plots will be used to assess reported bias when more than 10 trials are included. Its symmetry explains these deviations. If the funnel plot is symmetrical, no bias is reported, while asymmetry implies the presence of bias.

### 2.9. Data synthesis

The synthesis will be performed by generating a forest plot for meta-regression. If the heterogeneity test indicated that there was no substantial heterogeneity between studies, the Mantel-Haenszel method was fitted to calculate pooled estimates, 95% CIs, and combined *P* values. If substantial heterogeneity is indicated by *I*^2^ < 50%, the random effects model will be performed using the DerSimonian and Laird method (DerSimonian, 1986) and the rma function. The significance of the *P* value represents the strength of evidence against the null hypothesis of no intervention effect. In addition, the random effects variance and inconsistency variance were roughly equal, which is considered to be less inconsistent.

### 2.10. Subgroup analysis

We will perform a subgroup analysis to explore different types of moxibustion, treatment timing, and methodological quality when heterogeneity is high.

### 2.11. Sensitivity analysis

In order to test the robustness of the main decisions in the review process, we will conduct a sensitivity analysis. The main analysis points include the impact of method quality, sample size, and missing data on the study. The meta-analysis will be reused, and more inferior quality studies will be excluded. The results will be compared and discussed according to the results.^[15]^

### 2.12. Grading the quality of evidence

The quality will be evaluated by using the grading of recommendations assessment, development, and evaluation.^[16,17]^ The assessment results will be divided into 4 levels: very low, low, moderate, and high.

## 3. Discussion

Uterine contraction pain is being common among women in labor, with up to 60% of patients suffering from poor uterine regeneration and retained malodex.^[18]^ Nowadays, many studies have shown the advantages of moxibustion in the treatment of uterine contraction pain, which can promote postpartum uterine recovery and improve maternal quality of life.^[19]^ To the best of our knowledge, there has not been a meta-analysis on moxibustion for uterine contraction pain. A meta-analysis on the clinical situation of moxibustion for postpartum abdominal pain would provide a basis for clinical treatment. Therefore, we decided to conduct the present study to compare the efficacy and safety of moxibustion on uterine contraction pain. Our study will provide clinicians and guideline makers with the available evidence on non-pharmacological interventions. The results of this protocol will be published in relevant journals as soon as possible and will be updated rapidly in real time.

## Author contributions

XZ and BL had the original idea of this work and drafted the protocol. The search strategy was developed by all the authors and will be performed by XZ and BL, QZ, and HW independently screen the potential studies, extract data from the included studies, assess the risk of bias and complete the data synthesis. YT will arbitrate in cases of disagreement and ensure the absence of errors. All authors approved the publication of the protocol.

**Conceptualization:** Xuewei Zhao, Baiyan Liu.

**Data curation:** Qi Zhang, He Wang.

**Formal analysis:** Xuewei Zhao.

**Funding acquisition:** Fuchun Wang.

**Investigation:** Xuewei Zhao.

**Methodology:** Baiyan Liu, Yu Tian.

**Supervision:** Fuchun Wang.

**Validation:** Xuewei Zhao, Yu Tian.

**Writing – original draft:** Xuewei Zhao, Baiyan Liu.

**Writing – review & editing:** Xuewei Zhao, Fuchun Wang.
